# Maternal Administration of Probiotic or Prebiotic Prevents Male Adult Rat Offspring against Developmental Programming of Hypertension Induced by High Fructose Consumption in Pregnancy and Lactation

**DOI:** 10.3390/nu10091229

**Published:** 2018-09-04

**Authors:** Chien-Ning Hsu, Yu-Ju Lin, Chih-Yao Hou, You-Lin Tain

**Affiliations:** 1 Department of Pharmacy, Kaohsiung Chang Gung Memorial Hospital, Kaohsiung 833, Taiwan; chien_ning_hsu@hotmail.com; 2 School of Pharmacy, Kaohsiung Medical University, Kaohsiung 807, Taiwan; 3 Department of Obstetrics and Gynecology, Kaohsiung Chang Gung Memorial Hospital and Chang Gung University College of Medicine, Kaohsiung 833, Taiwan; lyu015@cgmh.org.tw; 4 Department of Seafood Science, National Kaohsiung University of Science and Technology, Kaohsiung City 811, Taiwan; chihyaohou@webmail.nkmu.edu.tw; 5 Department of Pediatrics, Kaohsiung Chang Gung Memorial Hospital and Chang Gung University College of Medicine, Kaohsiung 833, Taiwan; 6 Institute for Translational Research in Biomedicine, Kaohsiung Chang Gung Memorial Hospital and Chang Gung University College of Medicine, Kaohsiung 833, Taiwan

**Keywords:** fructose, hypertension, gut microbiota, short-chain fatty acid, nutrient-sensing signal, sensory receptor

## Abstract

Excessive intake of fructose is associated with hypertension. Gut microbiota and their metabolites are thought to be associated with the development of hypertension. We examined whether maternal high-fructose (HF) diet-induced programmed hypertension via altering gut microbiota, regulating short-chain fatty acids (SCFAs) and their receptors, and mediating nutrient-sensing signals in adult male offspring. Next, we aimed to determine whether early gut microbiota-targeted therapies with probiotic *Lactobacillus casei* and prebiotic inulin can prevent maternal HF-induced programmed hypertension. Pregnant rats received 60% high-fructose (HF) diet, with 2 × 10^8^ CFU/day *Lactobacillus casei* via oral gavage (HF+Probiotic), or with 5% *w/w* long chain inulin (HF+prebiotic) during pregnancy and lactation. Male offspring (*n* = 7–8/group) were assigned to four groups: control, HF, HF+Probiotic, and HF+Prebiotic. Rats were sacrificed at 12 weeks of age. Maternal probiotic *Lactobacillus casei* and prebiotic inulin therapies protect against hypertension in male adult offspring born to fructose-fed mothers. Probiotic treatment prevents HF-induced hypertension is associated with reduced plasma acetate level and decreased renal mRNA expression of *Olfr78*. While prebiotic treatment increased plasma propionate level and restored HF-induced reduction of *Frar2* expression. Maternal HF diet has long-term programming effects on the adult offspring’s gut microbiota. Probiotic and prebiotic therapies exerted similar protective effects on blood pressure but they showed different mechanisms on modulation of gut microbiota. Maternal HF diet induced developmental programming of hypertension, which probiotic *Lactobacillus casei* or prebiotic inulin therapy prevented. Maternal gut microbiota-targeted therapies could be reprogramming strategies to prevent the development of hypertension caused by maternal consumption of fructose-rich diet.

## 1. Introduction

Consumption of daily fructose from high-fructose corn syrup and refined sugar has been on the rise over the past several decades [1]. High-fructose (HF) diet in pregnancy has been shown to cause adverse effects on pregnancy and postnatal life [2,3]. Epidemiology suggests that excessive intake of fructose is associated with numerous common diseases, including hypertension [4]. Hypertension may originate in early life. The kidneys play a central role in controlling blood pressure (BP). During kidney development, maternal diet and early-life nutrition can result in long-lasting effects on renal function and structure, which is called renal programming [5], leading to increase the risk of developing hypertension in adulthood. This notion has become recognized as the developmental origins of health and disease (DOHaD) [6]. Our previous report demonstrated that HF diet during pregnancy and lactation produced hypertension in adult offspring [7]. Conversely, the DOHaD paradigm provides an opportunity to create reprogramming strategies to shift therapeutic interventions from adulthood to fetal life, before hypertension is evident [8].

Accumulating evidence reveals that dysbiosis of gut microbiota is implicated in the pathogenesis of hypertension [9,10,11,12]. Gut microbiota composition is altered after hypertension and that also gut microbiota has a role to play in the development of hypertension [11,12]. Conversely, gut microbiota-targeted therapies with probiotics and prebiotics have been suggested to be beneficial in hypertension and kidney disease [13]. Gut microbes metabolize dietary fibers to generate short-chain fatty acids (SCFAs). Evidences indicate that SCFAs are involved in BP control via interacting with sensory receptors, like metabolite-sensing G protein-coupled receptors (GPCRs) and olfactory receptors [14,15]. SCFAs such as acetate, propionate, and butyrate are vasodilatory properties in vitro. In spontaneously hypertensive rat (SHR), decreases in acetate- and butyrate-producing bacteria were relevant to hypertension [9]. Additionally, maternal nutritional insults may influence nutrient-sensing signals that leads to developmental programming of hypertension [16,17]. These signals are expressed in the kidney, including cyclic adenosine monophosphate-activated protein kinase (AMPK), silent information regulator transcript (SIRT), peroxisome proliferator-activated receptors (PPARs), mammalian target of rapamycin (mTOR), and PPARγ coactivator-1α (PGC-1α).

Despite recent studies showing a link between fructose-rich diet and dysbiosis of gut microbiota [18,19], data are lacking on the reprogramming effects of probiotics and prebiotics on the developmental programming of hypertension induced by maternal HF consumption. Our objective in this study was first to determine whether maternal HF diet-induced programmed hypertension via altering gut microbiota, regulating SCFAs and their receptors, and mediating nutrient-sensing signals in the kidney. The second aim was to determine whether early gut microbiota-targeted therapies with probiotic *Lactobacillus casei* or prebiotic inulin can be reprogramming strategies to prevent maternal HF-induced programmed hypertension in adult offspring.

## 2. Materials and Methods 

### 2.1. Animal Models

The Institutional Animal Care and Use Committee at the Kaohsiung Chang Gung Memorial Hospital approved the study protocol and all procedures (IACUC permit number: 2017031603). All animal experiments conformed to the Guide for the Care and Use of Laboratory Animals of the National Institutes of Health. Virgin Sprague Dawley (SD) rats (12–16 weeks old, *n* = 12) were purchased from BioLASCO Taiwan Co., Ltd. (Taipei, Taiwan). Rats were maintained in an Association for Assessment and Accreditation of Laboratory Animal Care International (AAALAC)-approved animal facility in our hospital with controlled temperature and 12:12 h light/dark cycles. Male SD rats were caged with individual females until mating was confirmed by observation of a vaginal plug. 

Pregnant SD rats were randomly allocated into four groups (*n* = 3/group) and fed as follows: (1) control group received regular chow (Fwusow Taiwan Co., Ltd., Taichung, Taiwan) during gestation and lactation, (2) HF group received chow supplemented 60% high-fructose (HF) diet (Harlan Teklad, Madison, WI, USA) during the entire gestation and lactation periods, (3) HF+Prebiotic group received 60% HF diet plus 5% *w/w* long chain inulin (Sigma, St. Louis, MO, USA) during gestation and lactation periods (i.e., a total of six weeks), and (4) HF+Probiotic group received 60% HF diet plus 2 × 10^8^ CFU/day *Lactobacillus casei* (Antibiophilus, Laboratoires Lyocentre, France) throughout gestation and lactation via gavage of 1 mL of prepared spore suspension using a blunt ended needle. The doses of prebiotics and probiotics used here are based on previous studies conducted in rats [20,21,22]. As men are more prone to hypertension at a younger age [23], only male offspring from litters that were culled to eight pups after birth were used in subsequent experiments.

BP-2000 tail-cuff system (BP-2000, Visitech Systems, Inc., Apex, NC, USA) was used in conscious rats for measurement of BP [7]. To ensure accuracy and reproducibility, the rats were acclimated to restraint and tail-cuff inflation for one week before the experiment. Three stable measurements were obtained and were averaged. Male offspring (*n* = 7–8/group) were sacrificed at 12 weeks of age. Heparinized blood samples were collected. The kidneys were harvested and stored at −80 °C freezer for further analysis.

### 2.2. Gas Chromatography-Flame Ionization Detector (GC-FID)

Plasma acetate, butyrate, and propionate levels were measured using gas chromatography-mass spectrometry (GCMS-QP2010; Shimadzu, Kyoto, Japan) with flame ionization detector (FID). Analytical standard grades used as internal standards for acetate and propionate were obtained from Sigma-Aldrich (St. Louis, MO, USA) and for butyrate was from Chem Service (West Chester, PA, USA). The working solutions of acetate, butyrate, and propionate used as internal and external standards were at the concentration of 10 mM and kept at −20 °C freezer. Dry air, nitrogen, and hydrogen were supplied to the FID at 300, 20 and 30 mL/min, respectively. An aliquot of 2 µL sample was injected into the column. The inlet and FID temperature were set at 200 and 240 °C, respectively. The total running time was 17.5 min. 

### 2.3. Gut Microbiota Profiling

Metagenomic DNA was isolated from frozen fecal samples were after centrifugation. Bacterial DNA was extracted and then amplified using specific forward and reverse primers as follows: 5′-TCGTCGGCAGCGTCAGATGTGTATAAGAGACAGCCTACGGGNGGCWGCAG-3′ and 5′-GTCTCGTGGGCTCGGAGATGTGTATAAGAGACAGGACTACHVGGGTATCTAATCC-3′ respectively, which targeted the V3-V4 region of the bacterial 16S rRNA gene. Amplicons were prepared according to the 16S Metagenomics Sequencing Library Preparation protocol (Illumina, San Diego, CA, USA), and sequenced with the Illumina MiSeq platform (Illumina, San Diego, CA, USA) in paired-end mode with 600-cycle sequencing reagent.

Next generation sequencing data were analyzed with the Microbial Genomics Module of CLC Genomics Workbench 9.5.4 (Qiagen, Stockach, Germany). Taxonomic relative abundance profiles (e.g., phylum, family, genus, and species) were compared using the Student’s *t*-test for independent samples. Linear discriminant analysis (LDA) effect size was used to agnostically identify microbial biomarkers. The LDA score represents the differences in genus-level abundance between grouping categories (e.g., control vs. HF). Microbial biomarkers in the current study were retained if they have a stringent cutoff value (LDA score log10 > 1).

### 2.4. Quantitative Real-Time Polymerase Chain Reaction (PCR)

RNA was extracted using TRIzol reagent treated with DNase I (Ambion, Austin, TX, USA) to remove DNA contamination, and reverse transcribed with random primers (Invitrogen, Carlsbad, CA, USA) [24]. RNA concentration and quality were checked by measuring optical density at 260 nm and A280 nm wavelengths. The complementary DNA (cDNA) product was synthesis using a MMLV Reverse Transcriptase (Invitrogen). Two-step quantitative real-time PCR was conducted using the QuantiTect SYBR Green PCR Kit (Qiagen, Valencia, CA, USA) and the iCycler iQ Multi-color Real-Time PCR Detection System (Bio-Rad, Hercules, CA, USA). Several genes related to the nutrient sensing signaling pathways were analyzed in this study, including AMP-activated protein kinase, subunit-α2 (*Prkaa2*), -β2 (*Prkab2*), and -γ2 (*Prkag2*); sirtuin-4 (*Sirt4*); peroxisome proliferator-activated receptor (PPAR)-α (*Ppara*), -β (*Pparb*), and -γ (*Pparg*); and PPARγ coactivator 1-α (*Ppargc1a*). Three genes, *Ffar3* (encodes for GPR41), *Ffar2* (encodes for GPR43), and *Olfr78* (encodes for olfactory receptor 78) belonging to sensory receptors were analyzed. We used 18S rRNA (*r18S*) as a reference. Primers were designed using GeneTool Software (BioTools, Edmonton, AB, Canada) (Table 1). All samples were run in duplicate. The comparative threshold cycle (CT) method was used to calculate relative gene expression. For each sample, the average CT value was subtracted from the corresponding average r18S value, calculating the ΔCT. ΔΔCT was calculated by subtracting the average control ΔCT value from the average experimental ΔCT. The fold-increase of the experimental sample relative to the control was calculated using the formula 2^−ΔΔCT^.

### 2.5. Western Blot

Western blot analysis was performed using the methods published previously [19]. We used the following primary antibodies: a goat polyclonal anti-rat AMPKα2 antibody (sc-19131, 1:1000, overnight incubation; Santa Cruz Biotechnology, Santa Cruz, CA, USA), a rabbit polyclonal anti-rat phosphorylated AMPKα1/2 antibody (sc-33524, 1:1000, overnight incubation; Santa Cruz Biotechnology), a rabbit polyclonal anti-rat mTOR antibody (Cell Signaling #2972, 1:1000, overnight incubation; Cell Signaling, Danvers, MA, USA) and a rabbit polyclonal anti-rat phosphorylated mTOR antibody (Cell Signaling #2971, 1:1000, overnight incubation; Cell Signaling). Bands were detected using SuperSignal West Pico reagent (Pierce, Rockford, IL, USA). The protein abundance was quantified by densitometry (Quantity One Analysis software; Bio-Rad, Hercules, CA, USA). The densitometer readings were expressed as integrated optical density (IOD), normalized to Ponceau S (PonS) staining to correct for variations in total protein loading and for an internal standard. Protein abundance was calculated as IOD/PonS.

### 2.6. Statistical Analysis 

Statistical analysis was conducted with one-way analysis of variance (ANOVA) with an LSD post hoc test for multiple comparisons. Data are reported as the mean ± standard error of mean (SEM) with a *p* value less than 0.05 being considered statistically significant. Analyses were performed using the Statistical Package for the Social Sciences (SPSS) software (Chicago, IL, USA).

## 3. Results

### 3.1. Morphological Features and Blood Pressures

**Figure 1:**
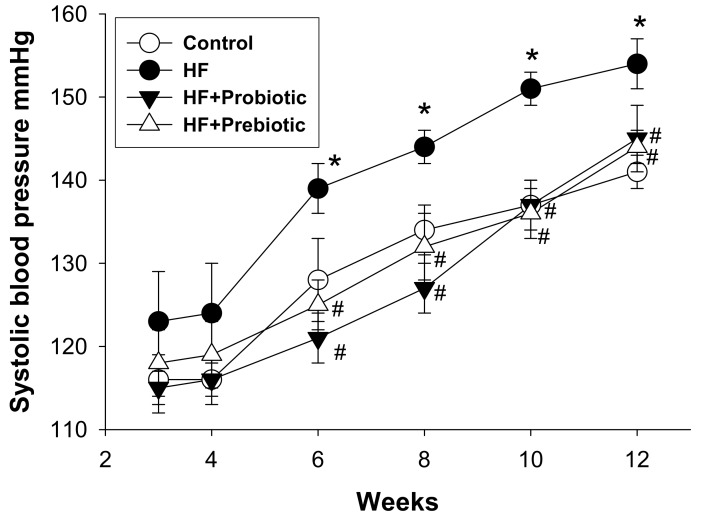
Effect of maternal high-fructose (HF) diet, probiotic *Lactobacillus casei*, and prebiotic inulin on systolic blood pressure in male offspring from 3 to 12 weeks of age. *N* = 7–8/group. * *p* < 0.05 versus control; # *p* < 0.05 versus HF.

The kidneys play a decisive role in the regulation of BP. Therefore, our work was mainly focused on the kidney. Table 2 shows that Body weight and kidney weight-to-BW ratio were lower in HF+Prebiotic group compared to other three groups. As shown in Figure 1, systolic blood pressure (SBP) was similar in the four groups at four weeks of age. The SBP of HF group was significantly greater than that of the control from 6 to 12 weeks of age during the development of hypertension. A similar reduction in SBP (~10 mmHg) was measured in the HF+Probiotic and HF+Prebiotic groups versus the HF group at 12 weeks of age (Table 2).

### 3.2. Short Chain Fatty Acids and Nutrient-Sensing Signals

It was reported previously that SCFAs and nutrient-sensing signals are involved in the development of hypertension [16,17,18,19]. We investigated whether maternal HF diet impairs SCFAs production while probiotic or prebiotic therapy prevents it. The results of the current study showed that HF diet significantly increased plasma level of acetate compared to control, which was restored by probiotic therapy (Table 3). Plasma butyrate level was not different among four groups. However, plasma level of propionate was highest in the HF+Prebiotic group among four groups. 

**Figure 2:**
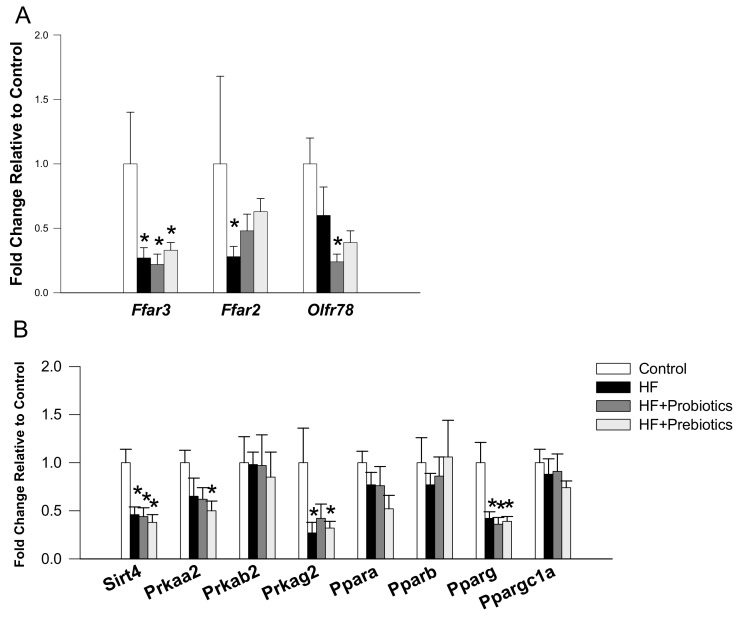
Effect of maternal high-fructose (HF) diet, probiotic *Lactobacillus casei*, and prebiotic inulin on gene expression of (**A**) SCFA receptors and (**B**) nutrient-sensing signaling pathway in offspring kidneys at 12 weeks of age. *N* = 7–8/group. * *p* < 0.05 versus control.

We next measured renal mRNA levels of SCFA receptors and genes involved in nutrient-sensing signaling pathway. Maternal HF diet significantly decreased renal mRNA expression of *Ffar3* (fold change (FC) = 0.27) and Ffar2 (FC = 0.28) compared with that in control, while the reduction of *Ffar2* expression was partially prevented by prebiotic (FC = 0.48) or probiotic therapy (FC = 0.63) (Figure 2A). We observed that renal mRNA expression of *Olfr78* (FC = 0.24) was lower in the HF+Probiotic group than that in the control group. Additionally, maternal HF diet decreased renal mRNA expression of several genes belonging to nutrient-sensing signaling pathway, including *Sirt4* (FC = 0.46), Prkag2 (FC = 0.27), and *Pparg2* (FC = 0.42). Renal mRNA expression of *Prkab2*, *Ppara*, *Pparb*, and *Ppargc1a* did not differ among the four groups (Figure 2B). However, renal mRNA expression of *Sirt4* (FC = 0.38), *Prkaa2* (FC = 0.5), *Prkag2* (FC = 0.32), and *Pparg2* (FC = 0.39) was reduced by HF+Prebiotic exposure.

**Figure 3:**
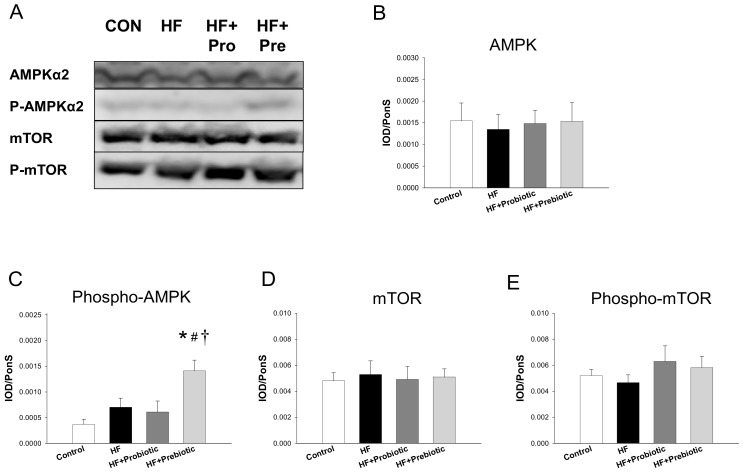
(**A**) Representative western blots showing AMPKα2 (~63 kDa), phosphorylated AMPKα2 (~63 kDa), mTOR (~289 kDa), and phosphorylated mTOR (~289 kDa) bands in offspring kidneys with maternal high-fructose (HF) diet and/or treatment with probiotic *Lactobacillus casei* and prebiotic inulin. Relative abundance of renal cortical (**B**) AMPKα2, (**C**) phosphorylated AMPKα2, (**D**) mTOR, and (**E**) phosphorylated mTOR were quantified. *N* = 7–8/group. * *p* < 0.05 versus control; # *p* < 0.05 versus HF; † *p* < 0.05 versus HF+Probiotic.

Maternal HF diet and probiotic/prebiotic therapies have no effect on renal protein levels of AMPKα2 (Figure 3B), mTOR (Figure 3D), and phosphorylated mTOR (Figure 3E). Nevertheless, maternal prebiotic therapy significantly increased renal protein level of phosphorylated AMPKα2 in offspring kidneys (Figure 3C). 

### 3.3. Gut Microbiota Profiling

**Figure 4:**
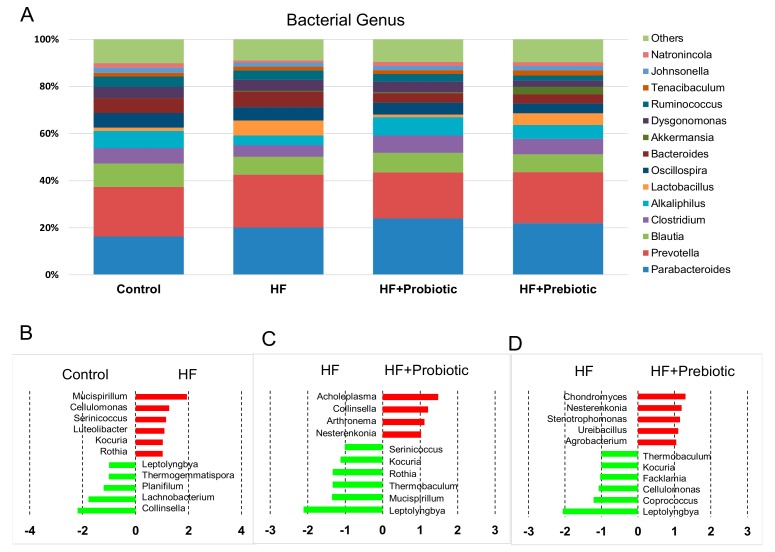
Effect of maternal high-fructose (HF) diet, probiotic *Lactobacillus casei*, and prebiotic inulin on gene expression of (**A**) relative abundances of the gut microbiota at the genus level. Linear discriminant analysis (LDA), along with effect size measurements, was applied to identify enriched bacterial genera. Most enriched and depleted genera (LDA score (log10) > 1.0) in the (**B**) HF (red) vs. control (green), (**C**) HF+Probiotic (red) vs. HF (green), and (**D**) HF+Prebiotic (red) vs. HF (green). *N* = 7–8/group.

**Figure 5:**
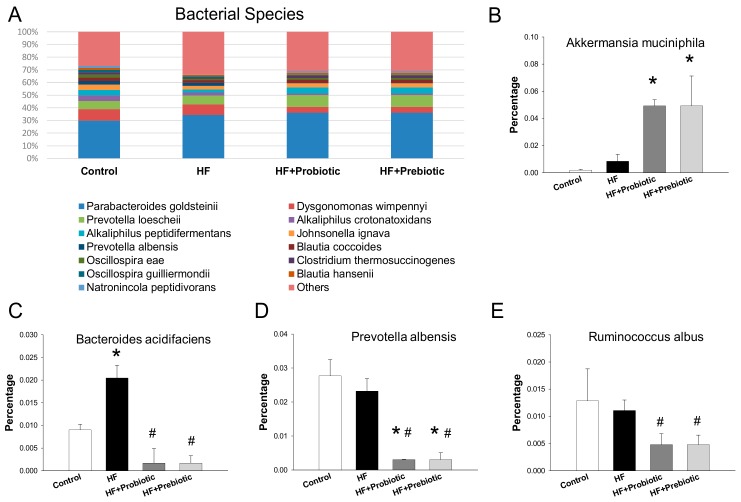
Effect of maternal high-fructose (HF) diet, probiotic *Lactobacillus casei*, and prebiotic inulin on gene expression of (**A**) Relative abundances of the gut microbiota at the species level; (**B**) the *Akkermansia muciniphila*; (**C**) the *Bacteroides acidifaciens*; (**D**) the *Prevotella albensis*; and (**E**) the *Ruminococcus albus*. *N* = 7–8/group. * *p* < 0.05 versus control; # *p* < 0.05 versus HF.

We further analyzed bacterial populations in the gut at the phylum, genus (Figure 4), and species levels (Figure 5), to determine the role of the gut microbiota in hypertension of developmental origin in response to HF, probiotic, and prebiotic treatments. We observed that the main phyla in the four groups studied were *Bacteroidetes*, *Firmicutes*, *Verrucomicrobia*, *Proteobacteria*, and *Actinobacteria*. There was a remarkable decrease in Actinobacteria in the HF+Prebiotic (0.29 ± 0.02%; *p* < 0.05) and HF+Probiotic group (0.29 ± 0.05%; *p* < 0.05) compared to the control (0.88 ± 0.24%). Additionally, we observed a decrease in the *Actinobacteria* to *Firmicutes* ratio in offspring exposed to HF+Probiotic (0.006 ± 0.001; *p* < 0.05) and HF+Prebiotic (0.008 ± 0.002; *p* < 0.05) vs. the control (0.021 ± 0.012). Results of a previous study reported that the *Firmicutes* to *Bacteroidetes* ratio might act as a microbial marker for gut dysbiosis and hypertension [9]. However, in the current study the *Firmicutes* to *Bacteroidetes* ratio was comparable between the four groups (Control = 0.77 ± 0.14; HF = 0.64 ± 0.19; HF+Probiotic = 0.68 ± 0.1; HF+Prebiotic = 0.72 ± 0.37).

The main bacterial genera in the four groups were *Parabacteroides*, *Prevotella*, *Blautia*, *Clostridium*, *Alkaliphilus*, *Lactobacillus*, *Oscillospira*, *Bacteroides*, and *Akkermansia* (Figure 4A). Probiotic and prebiotic therapy both increased abundance of genus *Parabacteroides* (HF+Probiotic: 23.9 ± 0.9%; HF+Prebiotic: 22 ± 1.5% vs. control: 16.3 ± 1.6%; both *p* < 0.05). Conversely, abundance of genus *Bacteroides* was reduced by probiotic (HF+Probiotic: 4.1 ± 0.5%) and prebiotic therapy (HF+Prebiotic: 4 ± 0.4%) compared with that in control (6.2 ± 0.6%; both *p* < 0.05). Maternal HF diet decreased abundance of genus *Alkaliphilus* (4.2 ± 0.3% vs. control: 7.3 ± 0.9%; *p* < 0.05), whereas the abundance of genus *Lactobacillus* was increased (6.3 ± 2.1% vs. control: 1.4 ± 0.3%; *p* < 0.05). These changes were restored by probiotic therapy. Additionally, the *Bacteroides* to *Prevotella* ratio was lower in the HF+probiotic (21 ± 2.3%) and HF+prebiotic group (20.2 ± 2.6%) compared with that in the control group (29.4 ± 1.8%; both *p* < 0.05).

The main bacterial genera modified by the maternal HF diet were *Mucispirillum* (LDA score = 1.9), *Lachnobacterium* (LDA score = −1.8), and *Collinsella* (LDA score = −2.2) (Figure 4B). Probiotic treatment showed an increase in genus *Acholeplasma* (LDA score = 1.5) and a decrease in genus *Leptolyngbya* (LDA score = −2.1) compared to that in the HF group (Figure 4C). Similarly, there was a remarkable decrease in the genus *Leptolyngbya* (LDA score = −2.1) in the HF+Prebiotic group vs. HF group (Figure 4D).

The main bacterial species in the four groups were shown in Figure 5. It is worth noting that maternal probiotic and prebiotic therapies significantly increased *Akkermansia muciniphila* in the HF+Probiotic (4.93 ± 0.45%) and HF+Prebiotic group (4.93 ± 2.21%) compared to the control group (0.19 ± 0.06%, both *p* < 0.05) (Figure 5B). *Bacteroides acidifaciens* was found increased in HF group and this increase was reverted in HF groups treated with probiotic or prebiotic therapy (Figure 5C). Additionally, abundance of *Prevotella albensis* and *Ruminococcus albus* were reduced by probiotic and prebiotic treatments vs. the HF group (Figure 5D,E). 

## 4. Discussion

This study provides a new insight into the mechanisms responsible for the maternal high-fructose diet-induced programmed hypertension with particular emphasis on SCFAs and their receptors, gut microbiota, and nutrient-sensing signals. The main novel findings in this study are: (1) maternal probiotic *Lactobacillus casei* or prebiotic inulin therapy protects against hypertension in male adult offspring of HF diet-fed dams; (2) maternal HF-induced programmed hypertension is relevant to increased plasma acetate level, decreased renal mRNA expression of *Frar3* and *Frar2*, and decreased several nutrient-sensing signals (i.e., *Sirt4*, *Prkag2*, and *Pparg2*); (3) probiotic treatment prevents HF-induced hypertension is related with reduced plasma acetate level and decreased renal mRNA expression of *Olfr78*; (4) prebiotic treatment increases plasma propionate level and restores HF-induced decreased *Ffar2* expression; (5) maternal HF diet has long-term programming effects on the adult offspring’s gut microbiota, including decreases abundance of genus *Alkaliphilus* and increases the abundance of genus *Lactobacillus*; (6) probiotic and prebiotic therapy show the similar BP-lowering effects but they have quite different mechanisms on modulation of gut microbiota. 

The present study is consistent with previous reports showing that consumption of various degrees of high-fructose diet ranging from 10–60% by pregnant dams leads to programmed hypertension in their adult offspring [3,7,25]. Certain probiotic strains like *Lactobacillus* and prebiotics have shown hypotensive effects [10,11]. Despite a meta-analysis of 702 individuals reported that probiotic fermented milk has BP-lowering effects in adults with prehypertension or hypertension [26], little is known about reprogramming effects of probiotics on the developmental programming of hypertension. The antihypertensive effect of either probiotic or prebiotic therapy was starting from six weeks of age (i.e., three weeks after stopping probiotic or prebiotic therapy) and over time. These findings suggest that the reduction of BP is due to reprogramming effect instead of an acute effect. We provide the first evidence, to our knowledge, that early probiotic therapy with *Lactobacillus casei* prevents maternal HF-induced programmed hypertension in adult offspring. Similar to probiotic therapy, our study also demonstrates that maternal prebiotic inulin therapy protects against hypertension of developmental origins induced by maternal HF consumption. 

In the current study, HF-induced hypertension was related to alterations in plasma SCFAs and their receptors in adult offspring kidneys. The results of this study showed that maternal HF diet increased plasma acetate level and decreased renal mRNA expression of *Ffar3* (GPR41) and *Ffar2* (GPR43). Acetate is a ligand for *Olfr78* to raise BP [12]. This, in turn, can be counteracted by the vasodilatory action of GRP41 and GPR43 [12]. Our report showed that probiotic therapy with *Lactobacillus casei* lowered plasma acetate level, reduced renal mRNA expression of *Olfr78*, and restored the HF-induced reduction of *Ffar2* (GPR43) expression, all of which may hence contribute to the protective effect on maternal HF diet-induced programmed hypertension. Another SCFA, propionate, has been reported to induce vasodilatation via mediating GPR41 receptor [12]. We observed that plasma propionate level was induced significantly by prebiotic inulin therapy. In line with this, a previous study showed that inulin increased propionate production [27]. Thus, the reprogramming effects of prebiotic therapy may, at least in part, regulate propionate and its sensory receptors to protect adult offspring against maternal HF-induced programmed hypertension.

Our results are in agreement with those of previous studies showing that high fructose intake reduces nutrient-sensing signals [28,29]. Probiotic therapy has nearly no effect on renal nutrient-sensing signals at both mRNA and protein levels. Notably, maternal prebiotic therapy significantly increased phosphorylated AMPKα2 protein level, which supports the notion that AMPK activation protects against hypertension [30]. 

Additionally, the beneficial effects of probiotic and prebiotic therapies could be due to mediation of the composition of the gut microbiota. Although the *Firmicutes* to *Bacteroidetes* ratio was reported as a microbial marker for gut dysbiosis and hypertension [9], which was not supported by our results. It is noteworthy that maternal probiotic and prebiotic therapies similarly reduced phylum *Actinobacteria* abundance as well as the *Actinobacteria* to *Firmicutes* ratio. Since obesity, a common phenotype of high-fructose consumption, has been linked to a higher level of *Actinobacteria* phylum in both human and animal studies [31], additional study is required to clarify whether the *Actinobacteria* abundance and *Actinobacteria* to *Firmicutes* ratio may serve as a microbial marker for programmed hypertension in other DOHaD models. Additionally, probiotic or prebiotic therapy significantly increased the abundance of *Akkermansia muciniphila*, one of the beneficial gut propionate-producing microbes [32]. According to our data, probiotic or prebiotic therapy protects maternal HF diet-induced programmed hypertension related to decreased abundance of *Bacteroides acidifaciens*. Since *Bacteroides* has been presumed to represent as a biomarker of diet and lifestyle [33], our results suggest the potential role of *Bacteroides acidifaciens* as a microbial marker for programmed hypertension. It was reported that subjects with a low *Bacteroides* to *Prevotella* ratio appeared more susceptible to lose body fat on diets high in fiber [34]. Our data showed maternal probiotic or prebiotic therapy reduced *Bacteroides* to *Prevotella* ratio in adult offspring. Since maternal HF diet produced metabolic syndrome-related comorbidities [35], these findings suggest probiotic or prebiotic therapy might have some clinical relevance with regard to other phenotypes of metabolic syndrome, such as dyslipidemia and obesity. We found that maternal HF diet decreased *Collinsella* abundance, while prebiotic therapy increased it. Our results contradict a previous report showing that the abundance of the genus *Collinsella* positively correlate with insulin level in obese women [36]. Additionally, results from a previous study demonstrate that *Mucispirillum* is associated with a healthy gut [37]. However, we observed that maternal HF diet increased *Mucispirillum*, which was reversed by probiotic therapy. Hence, it is not known whether specific alterations of gut microbiota interact directly with hypertension phenotype and whether they act as a counterbalancing mechanism in response to maternal HF diet.

A potential limitation of this study is the inability to test different probiotic/prebiotic types, doses, and treatment durations. Second, we restricted probiotics or prebiotics therapy to the HF group. This was because in healthy people, probiotics/prebiotics have only minor adverse effect, if any. However, more research is needed to explore whether probiotics/prebiotics affect intestinal microbiota in normal control offspring. 

Furthermore, we analyzed gut microbiota in offspring at 12 weeks of age, the observed alterations might be a consequence of programmed hypertension. Although results of a previous study support alterations of gut microbiota happening as a result of hypertension [38], additional studies to analyze gut microbiota at different developmental windows might aid in identifying persistently programmed changes in response to early insults and exploring the causal relationship.

## 5. Conclusions

In summary, this is the first study that links the protective effects of maternal probiotic or prebiotic therapy on maternal HF-induced hypertension to SCFAs and their receptors, alterations of gut microbiota, and nutrient-sensing signals. Our data highlights that reprogramming strategies targeting the above-mentioned mechanisms that affect gut-microbiota and their metabolites could be developed for prevention of hypertension induced by widespread consumption of food and beverages with high fructose in pregnant mothers and their children.

## Figures and Tables

**Table 1:** Quantitative real-time polymerase chain reaction primers sequences.

Gene Sympol	Gene Name	Forward	Reverse
*Sirt4*	Sirtuin-4	5′-ccctttggaccatgaaaaga-3′	5′-cggatgaaatcaatgtgctg-3′
*Prkaa2*	AMP-activated protein kinase, subunit-α2	5′-agctcgcagtggcttatcat-3′	5′-ggggctgtctgctatgagag-3′
*Prkab2*	AMP-activated protein kinase, subunit-β2	5′-cagggccttatggtcaagaa-3′	5′-cagcgcatagagatggttca-3′
*Prkag2*	AMP-activated protein kinase, subunit-γ2	5′-gtgtgggagaagctctgagg-3′	5′-agaccacacccagaagatgc-3′
*Ppara*	Peroxisome proliferator-activated receptor-α	5′-agaagttgcaggaggggatt-3′	5′-ttcttgatgacctgcacgag-3′
*Pparrb*	Peroxisome proliferator-activated receptor-β	5′-gatcagcgtgcatgtgttct-3′	5′-cagcagtccgtctttgttga-3′
*Pparg*	Peroxisome proliferator-activated receptor-γ	5′-ctttatggagcctaagtttgagt-3′	5′-gttgtcttggatgtcctcg-3′
*Ppargc1a*	Peroxisome proliferator-activated receptor-γ coactivator 1-α	5′-cccattgagggctgtgatct-3′	5′-tcagtgaaatgccggagtca-3′
*Ffar3*	Free Fatty Acid Receptor 3	5′-tgaccatttcggacctgctt-3′	5′-tgggtaggctacgctcagaa-3′
*Ffar2*	Free Fatty Acid Receptor 2	5′-gctgtggtgttcagttccct-3′	5′-gtttgactcccacccctgtc-3′
*Olfr78*	Olfactory receptor 78	5′-accggtatgtggctatctgc-3′	5′-gtgggagagcacattggagt-3′
*R18S*	18S rRNA	5′-gccgcggtaattccagctcca-3′	5′-cccgcccgctcccaagatc-3′

**Table 2:** Summary of morphological values and blood pressure

	Control	HF	HF+Probiotic	HF+Prebiotic
	*N* = 8	*N* = 8	*N* = 7	*N* = 8
Body Weight (BW) (g)	452 ± 12	448 ± 10	457 ± 6	375 ± 3 *#†
Left Kidney Weight (g)	1.69 ± 0.06	1.76 ± 0.04	1.8 ± 0.04	1.68 ± 0.07
Left Kidney Weight/100 g BW	0.37 ± 0.01	0.39 ± 0.01	0.39 ± 0.01	0.45 ± 0.02 *#†
Systolic Blood Pressure (mmHg)	141 ± 1	154 ± 1 *	145 ± 2 #	144 ± 1 #

* *p* < 0.05 versus control; # *p* < 0.05 versus HF; † *p* < 0.05 versus HF. HF, mother rats received 60% high-fructose diet; HF+Probiotic, HF-treated mother rats received *Lactobacillus casei*; HF+Prebiotic, HF-treated mother rats received 5% inulin.

**Table 3:** Plasma levels of acetate, butyrate, and propionate

	Control	HF	HF+Probiotic	HF+Prebiotic
	*N* = 8	*N* = 8	*N* = 7	*N* = 8
Acetate (μM)	12.5 ± 0.4	20.3 ± 1.2 *	12.3 ± 1.1 #	18.6 ± 0.7
Butyrate (μM)	5.72 ± 0.38	8.56 ± 0.87	4.4 ± 0.72	7.01 ± 0.4
Propionate (μM)	0.82 ± 0.04	0.96 ± 0.08	0.87 ± 0.07	2.39 ± 0.3 *#†

* *p* < 0.05 versus control; # *p* < 0.05 versus HF; † *p* < 0.05 versus HF. HF, mother rats received 60% high-fructose diet; HF+Probiotic, HF-treated mother rats received *Lactobacillus casei*; HF+Prebiotic, HF-treated mother rats received 5% inulin.
